# Rwanda’s success in advancing midwifery education: a blueprint of a sustainable, nationally driven curriculum standardization

**DOI:** 10.1080/16549716.2024.2427467

**Published:** 2024-11-13

**Authors:** Malin Bogren, Menelas Nkeshimana, Innocent Nzabahimana, Frida Temple, Marie Claire Iryanyawera, Jean de Dieu Uwimana, Renata Tallarico, Olugbemiga Adelakin, Kerstin Erlandsson

**Affiliations:** a Institute of Health and Care Sciences, Sahlgrenska Academy, University of Gothenburg, Gothenburg, Sweden; b Ministry of Health, Health Workforce Development Department, Kigali, Rwanda; c United Nations Population Fund, Kigali, Rwanda; d Directorate of Teaching and Learning Enhancement, University of Rwanda, Kigali, Rwanda; e School of Health and Welfare, Dalarna University, Falun, Sweden

**Keywords:** Curriculum development, East Africa, midwife, midwifery education, stakeholder engagement

## Abstract

The International Confederation of Midwives (ICM) defines and sets the Essential Competencies for Midwifery Practice and provides a framework for developing and reviewing midwifery curricula. This framework ensures that pre-service midwifery education designed for students leads to the demonstration of the required midwifery specific competencies. The development of the ICM competencies in 2024 confirms the timeliness of the effort of Rwanda to update its national curricula. This commentary showcases the blueprint followed by Rwanda to standardize and culturally adapt its midwifery curricula at diploma, bachelor and master’s level to be competency-based and aligned with ICM. National ownership played a pivotal role in the standardization process, as the direction, priorities, and implementation of the curricula review initiative were driven by the country’s own government, higher learning institutes, national midwifery association and other national organizations. Rwanda’s experience in aligning its national curricula with international standards could serve as a model for south–south cooperation.

## Paper context

- **Main findings:** National ownership plays a key role in driving a standardization curriculum development process.
- **Added knowledge:** Rwanda’s experience demonstrates how national ownership can effectively drive curriculum reform by integrating international midwifery education standards into national standards while maintaining cultural relevance.
- **Global health impact for policy and action:** Rwanda’s experience in aligning its national curricula with international standards could serve as a model for south–south cooperation.

## Introduction

Midwives can make a significant contribution to the improvement of sexual, reproductive, and maternal health and reduction of mortality and morbidities, but only if they are adequately educated, regulated, well-integrated in the health systems, and supported by a professional organization [1]. The International Confederation of Midwives (ICM) clearly defines and sets out Essential Competencies for Midwifery Practice [2] and provides a framework for developing and reviewing midwifery curricula to ensure that pre-service midwifery education is designed for students to demonstrate midwifery-specific competencies. The recent development and release of these competencies in 2024 underscore the timely nature of Rwanda’s initiative to update its national midwifery curricula. Previous research has highlighted the importance of incorporating ICM competencies into midwifery curricula, particularly in low-income settings, illustrating the necessity of this process for aligning educational competencies with international standards [3].

Over the past 20 years, the Government of Rwanda achieved success in the reduction of maternal mortality from 1071 to 203 deaths per 100,000 live births [4], largely due to the expansion of the midwifery workforce among other strategies. However, the decline has slowed down in the last five years even though more than 93% of women now give birth in health facilities [5]. This poses a new challenge of how the country can sustain the progress made while maintaining high quality care.

The government recently launched the 4 × 4 reform [6], aiming to quadruple the current healthcare workforce within four years, with emphasis on educating midwives as one of the most pressing priorities. This reform includes investments in high-quality midwifery education, focusing on standardizing the midwifery curriculum and faculty development. The Ministry of Health in collaboration with the United Nations Population Fund (UNFPA) in Rwanda engaged the University of Gothenburg, Sweden, and a national curriculum consultant to support the curriculum standardization process. Working closely with a national core group representing eight higher learning institutes, the midwifery professional association, the regulatory body, and relevant international and national organizations, the Government conducted series of workshops from May to August 2024 to standardize and culturally adapt midwifery curricula.

The World Health Organization’s guide to developing high quality, sustainable midwifery education identifies key strategic priorities for midwifery education, faculty development and research. This included the need to align global education standards with national-level coordination among stakeholders [7]. This commentary therefore showcases the blueprint followed by Rwanda to standardize and culturally adapt midwifery curricula at the diploma, bachelor’s and master’s degree levels to be competency-based and aligned with the ICM Essential Competencies for Midwifery Practice.

### The process of standardizing midwifery curricula

#### Preparatory meetings

The initial phase involved a series of preparatory meetings to formalize the initiative and ensure a shared understanding of existing gaps in midwifery education and to clarify roles and responsibilities between the Ministry of Health, UNFPA, and the national and University of Gothenburg consultants. These discussions led to a concerted way forward, resulting in a clear timebound roadmap including agreement on workshops’ designs to ensure alignment with the initiative’s objectives.

*A 4-day Gap Analysis workshop* was conducted with around 30 midwifery leaders and educators from six higher learning institutes across the country and representatives from government, non-government, national and international organizations referred to as the core group to identify the competencies necessary for achieving the intended learning outcomes. The ICM Curriculum Mapping Tool was utilized. This analysis was further strengthened by comparing the existing national curricula with the national standards. The core group worked in pairs using a prepared matrix. The outcomes of these analyses are documented in an unpublished report that outlines the steps required to align the curricula with the ICM Essential Competencies for Midwifery Practice [2].

*A 5-day Alignment of Curricula workshop* focused on merging the existing midwifery curricula from different higher learning institutes and ensuring alignment with the ICM Essential Competencies for Midwifery Practice [2]. During this workshop, the templates provided by the Higher Education Council (HEC) for competency-based curriculum were used to ensure national adaptation. Furthermore, the progression and advancement of competencies in theory and clinical skills were articulated across different academic levels.

#### A 3-day quality assurance workshop

To ensure the quality of the curricula, a thorough review was conducted with the core group and specially invited education leaders to assess the overall structure; identify any wording difficulties; and enhance the appropriateness, feasibility, clarity, usability, and cultural relevance of the curricula content. Feedback was sought to guarantee alignment with the ICM Essential Competencies for Midwifery Practice [2], learners’ needs, and attainable competencies.

#### Online peer reviews

Beyond the face-to-face workshops, online peer reviews were conducted by the core group, in pairs, as a step to ensure that the midwifery curricula were fit for purpose and are robust enough to equip midwifery students with the competencies required for effective practice in Rwanda.

#### 1-day high-level validation and endorsement

Following the review by the core group and education leaders, the revised curricula were presented for endorsement to high-level authorities from relevant institutions, the Ministry of Health, Ministry of Education, higher learning institutes, and members of the core group. Thereafter, the revised curricula underwent a specific validation process by HEC to ensure their compliance with higher education standards and quality assurance guidelines in Rwanda.

#### 1-day high-level dissemination meeting

Finally, high-level stakeholder engagement was successfully carried out to secure endorsement of the final draft of the midwifery curricula. The curricula were then submitted to the relevant authorities within the government of Rwanda for final approval.

## How national ownership leads change

National ownership played a pivotal role in the standardization process by ensuring that the direction, priorities, and implementation of the curricula review initiative were driven by the country’s own government, higher learning institutes, national midwifery association and national organizations. This locally led approach brought several advantages:

- *Alignment with country’s specific needs and priorities*: When a change is led by national entities, it is considered more likely to be aligned with the specific needs, cultural contexts, and priorities of the country. This ensured that the process of standardization of the curricula remained relevant across the steps with a higher chance of success in its development and implementation.
- *Legitimacy and buy-in*: National ownership fostered trust and support among academic leaders and the midwifery faculty, leading to smoother and effective development and implementation.
- *Capacity building and adaptability*: Leading change at the national level encouraged the development of midwifery faculty capacities through active participation in workshops and peer reviews online. Additionally, it allowed for greater flexibility and responsiveness to changing circumstances due to the recently launched 4 × 4 reform [6]. This approach demonstrated how national ownership can catalyze successful and sustainable change in healthcare and higher education.
- *National accountability and empowerment*: Taking ownership of the change process made the Government of Rwanda accountable to its citizens to meet the objectives in the 4 × 4 reform [6]. This process provided another opportunity for the government to demonstrate, again, its ability to respond directly to the needs of various constituencies by leading a transparent, effective, and responsible governance approach that in this process fostered a sense of empowerment and pride among involved stakeholders.

## Next steps

High level evidence supports midwives’ role in providing universal health care and improving health outcomes for all [1]. Equally critical in improving health outcomes is ensuring that those who educate the next generation of midwives are well prepared and supported to deliver high-quality education [8,9]. Faculty development, which involves developing and strengthening educators, is a crucial strategy for improving the quality of care provided by maternity health professionals, ultimately reducing maternal and newborn mortality [1]. Considering this, efforts to standardize the midwifery curricula in Rwanda have resulted in a competency-based curricula at the diploma, bachelor’s, and master’s levels. The next step in delivering these curricula is to identify the needs of midwifery faculty to effectively implement these new competency-based education programmes. Additionally, supporting the government through the national regulatory mechanism in enforcing the implementation process will be vital for ensuring successful implementation.

The Alliance to Improve Midwifery Education (AIME) advocates for harnessing partnerships to strengthen global midwifery education [10]. We fully agree, emphasizing the importance of fostering partnerships at the national level, with Rwanda as an exemplary case. These national partnerships must include those involved in delivering education. Through in-country face-to-face workshops and meetings with faculty, there is potential to drive change at the national level. However, unless the national contextualization aligns with the ICM’s essential competencies for midwives [2] and the faculty is well-equipped to deliver this competency-based curriculum, improvements in health outcomes will be difficult to achieve. It is recommended that the ICM and AIME consider developing competencies on midwifery higher education, leadership and research at the master’s degree level. This initiative could be inspired by the Rwandan example, as such competencies are currently limited in the ICM’s essential competencies for midwifery practice [2].

## Conclusion

Rwanda’s nationally led efforts to advance midwifery education in line with the ICM Essential Competencies for Midwifery Practice have been successful by ensuring that the midwifery curricula review initiative was culturally appropriate and nationally led, built local capacities, and promotes accountability while ensuring sustainability in the long term. This process has not been without challenges. The accelerated timeline was challenging in balancing all tasks in alignment with the 4 × 4 strategy while considering the practicalities of curriculum development. While more time would have been beneficial, long timelines can also be counterproductive. The lesson is for other countries to carefully consider the ideal duration, learning from this experience. Rwanda is a pioneer in this process and could be a partner for south–south cooperation to support other countries that may be interested in undertaking the same journey.

## References

[1] Nove A, Friberg IK, de Bernis L, et al. Potential impact of midwives in preventing and reducing maternal and neonatal mortality and stillbirths: a lives saved tool modelling study. Lancet Global Health. 2021;9:e24–4. doi: 10.1016/s2214-109x(20)30397-1 Epub 2020/12/05. PubMed PMID: 33275948; PubMed Central PMCID: PMC7758876. 33275948 PMC7758876

[2] Midwives ICo. Essential competencies for midwifery practice 2024. Available from: https://internationalmidwives.org/resources/essential-competencies-for-midwifery-practice/

[3] Moller AB, Welsh J, Ayebare E, et al. Are midwives ready to provide quality evidence-based care after pre-service training? Curricula assessment in four countries-Benin, Malawi, Tanzania, and Uganda. PLOS Glob Public Health. 2022;2:e0000605. doi: 10.1371/journal.pgph.0000605 Epub 20220919. PubMed PMID: 36962507; PubMed Central PMCID: PMC10021168. 36962507 PMC10021168

[4] World Health Organization. Trends in maternal mortality 2000 to 2020: estimates by WHO, UNICEF, UNFPA, world bank group and UNDESA/Population division. Geneva: World Health Organization; 2023.

[5] National Institute of Statistics of Rwanda - NISR, Ministry of Health - MOH, ICF. Rwanda demographic and health survey 2019-20. Kigali, Rwanda and Rockville (MD), USA: NISR/MOH/ICF; 2021.

[6] Ministry of Health. The 4x4 reform: a path to quality health care in Rwanda. 2023.

[7] WHO. Strengthening quality midwifery education for universal health coverage 2030: framework for action. 2019. Geneva.

[8] West F, Homer C, Dawson A. Building midwifery educator capacity in teaching in low and lower-middle income countries. A review of the literature. Midwifery. 2016;33:12–23. doi: 10.1016/j.midw.2015.06.011 Epub 20150625. PubMed PMID: 26183921. 26183921

[9] UNFPA, ICM, WHO. The state of the world’s midwifery 2021. (NY): United Nations Population Fund; 2021.

[10] Bar-Zeev S, Shikuku D, Homer C, et al. Harnessing partnerships to strengthen global midwifery education to improve quality maternal and newborn health care: the alliance to improve midwifery education (AIME). Midwifery. 2024;137:104111. doi: 10.1016/j.midw.2024.104111 39127574

